# Indefinite Pain

**Published:** 1918-08

**Authors:** 


### ABSTRACTS

**Indefinite Pain.** Smith, E. B., Medical Council. Writing of pain, he says, "Pain can only be present where there is involvement of the sensory nervous system and is due to one of three conditions, or possibly at times all three included; injury, irritation or starvation. The first is usually evident to the naked eye; the second, if localized, may be also, but if central must come under the third reading, which can only be discovered by urinary examination." Much stress is laid on the old supposition of lithemia; an excess of uric acid or urates, as being the cause. He says, "This old theory should be abandoned, especially in view of the fact that the true condition can be discovered in a very few minutes by an estimation of the alkaline phosphates which show nerve-cell metabolism; this is easily accomplished by use of the Dowd Phosphatometer." Regarding irritation and starvation of the nerve cells, the condition that must be ascertained by alkaline estimation he says, "In fully 90 per cent. of these cases an index will be found divided about as follows: 75% due to starvation which call for nerve-cell nutrition, (Phosphorus he advises as the ideal remedy), and 15% to irritation, a condition (showing plus on the phosphatometer practically) always accompanied by high blood pressure, and calling for sedatives, as the bromide of gold and arsenic." He reports several cases in each group, which after months of treatment by other measures were not only relieved of indefinite pain, and when accompanied by high blood pressure, this, in a goodly number of cases, was reduced at once.

